# Pet Owners and Antibiotics: Knowledge, Opinions, Expectations, and Communication Preferences

**DOI:** 10.3390/antibiotics10111326

**Published:** 2021-10-29

**Authors:** Ri Scarborough, Laura Hardefeldt, Glenn Browning, Kirsten Bailey

**Affiliations:** 1 Asia-Pacific Centre for Animal Health, Department of Veterinary Biosciences, Melbourne Veterinary School, Faculty of Veterinary and Agricultural Sciences, University of Melbourne, Parkville, VIC 3010, Australia; laura.hardefeldt@unimelb.edu.au (L.H.); glenfb@unimelb.edu.au (G.B.); baileyk@unimelb.edu.au (K.B.); 2 National Centre for Antimicrobial Stewardship, Peter Doherty Institute, Parkville, VIC 3052, Australia

**Keywords:** antimicrobial, resistance, veterinary, dog, cat, owner, stewardship, survey, education, delayed prescribing

## Abstract

Despite the important role of antimicrobial use in companion animals in the global challenge presented by antimicrobial resistance (AMR), very few studies have quantified pet owner factors that can contribute to suboptimal veterinary antimicrobial use. We conducted an online survey of pet owners, asking about their experiences with veterinarians, their opinions on antibiotic use and knowledge of antibiotics, and their communication preferences regarding judicious prescribing. Just over half (54%) of the 558 pet owners had received antibiotics for their pet at their last non-routine veterinary consultation and most owners were happy (83%) with the antibiotic prescribing decision of their veterinarian. A quarter (25%) indicated that they had been surprised, disappointed or frustrated when a veterinarian had not given their pet antibiotics; 15% had explicitly requested them. Owners placed a higher priority on their pet receiving the most effective treatment than on treatment being cheap or convenient. Most respondents recognized the limitations of antibiotic therapy and the risks associated with antibiotic use, but 50% believed the risks were confined to the treated animal; only a minority was aware of inter-species transfer of bacteria. Pet owners indicated that they would find judicious prescribing messages focused on the direct risks of antibiotics to their pet more compelling than those about public health. Our findings suggest that veterinary communications about responsible antibiotic use should focus on pet owners’ priorities and address or bypass their gaps in understanding regarding antibiotic resistance.

## 1. Introduction

The preservation of the effectiveness of existing antimicrobial drugs, for all species, in the face of escalating bacterial antimicrobial resistance (AMR) is a shared responsibility, demanding effort from all sectors involved in the use of antimicrobial drugs.

There is significant overlap between the antimicrobial drugs used in companion animals and those used in humans. In addition, the popularity of companion animals and the physical closeness many people have with their pets as valued members of their families [1] increases the risk of antimicrobial-resistant bacteria, and resistance determinants, transferring from humans to pets, and vice versa.

In Australia, 61% households have a pet; 40% have at least one dog and 27% have at least one cat [1]. Legally, pets can only be given antimicrobial drugs on prescription from a registered veterinary practitioner, who also usually dispenses the drugs from their clinic. Compared with human medical practice, companion animal practice in Australia uses antibiotics relatively infrequently; antimicrobial exposure per life-year in dogs and cats is less half that of Australian people, and the proportion of dog and cat prescriptions representing the highest priority critically important antimicrobials (HPCIAs) is low [2]. While companion animal practitioners in several other countries use higher proportions of HPCIAs, particularly third-generation cephalosporins and fluoroquinolones [3,4,5,6,7,8], there is undoubtedly scope in all countries to improve antimicrobial use in companion animal practice [9].

Suboptimal antimicrobial use by prescribers can be conceptualized in two broad categories: firstly, using an antimicrobial drug where none is needed, for example, antibiotics for a viral infection, or for a bacterial infection which can usually be eliminated without antimicrobials (e.g., cat fight abscess); and secondly, where antimicrobials are indicated but the prescriber chooses a suboptimal drug, dose, timing, duration or route of administration. Expectations of pet owners that their pet should receive medication, often specifically antimicrobial drugs, combined with a lack of understanding of the risks associated with antimicrobial drugs, have been identified as drivers of suboptimal antimicrobial prescribing in companion animals [10,11,12,13,14,15]. The availability of the long-acting HPCIA injection cefovecin (Convenia) for dogs and cats presents particular challenges for veterinarians. As a third-generation cephalosporin, it carries a high risk of selecting for resistant bacteria, including those producing extended-spectrum beta lactamases (ESBLs), and its Australian product information states that it should only be used ‘where indicated by antibiotic sensitivity testing, according to principles of prudent use’ [16]. However, there is robust evidence that it is almost always administered without prior antibiotic sensitivity testing [17,18] and, anecdotally, is requested by some pet owners because of the ease of its administration compared with (more appropriate) oral antimicrobials.

Simple antimicrobial stewardship (AMS) interventions to tackle drivers of inappropriate use could include education of pet owners about antimicrobial resistance–for example, through informative posters in clinic waiting rooms–and training for veterinarians in communication skills. It is critical that such education and communication skills training are tailored to the knowledge and attitudes of pet owners with respect to antimicrobials for their pets [19]. We identified only two previous quantitative studies of such factors in pet owners internationally: a discrete choice survey of dog owners, primarily from North America [20], which elicited the priorities of dog owners when choosing between two antimicrobials; and a survey of cat owners in the United Kingdom (UK) [21], exploring attitudes to and knowledge about antimicrobials. Four qualitative studies have thematically documented a range of pet owner views of antibiotics in the UK [14,15,22] and the United States of America (USA) [23], but no studies of Australian pet owners have been published. No studies to date have examined the preferences of pet owners regarding how veterinarians communicate when declining or delaying antimicrobial therapy, or when steering owners towards a more appropriate, but less convenient, treatment, despite the importance of these conversations for antimicrobial stewardship in companion animal medicine. The influence of pet owner characteristics on opinions, knowledge and preferences with respect to antimicrobial use has undergone very little examination.

The primary aim of this cross-sectional survey study was to investigate the opinions, expectations and preferences of cat and dog owners with respect to veterinary consultations and antimicrobial drugs, and to evaluate their understanding of the risks associated with antimicrobial use. The secondary aim was to determine whether there were differences in responses between demographic groups, including age, socio-economic advantage, and educational background. The goal was to provide a basis for veterinarians to increase the effectiveness of their conversations with pet owners and inform future AMS interventions in companion animal veterinary practice.

## 2. Results

### 2.1. Respondents

A total of 662 responses were received. One hundred and four participants provided responses to fewer than 70% of questions, including key questions about attitudes and knowledge. These responses were disregarded and the remaining 558 responses were analysed. The demographics of participants whose responses were excluded were similar to those of the participants whose responses were included (Figure S1, Supplementary Materials), suggesting that there were non-systematic reasons for the failure of some participants to complete the survey.

**Figure 1:**
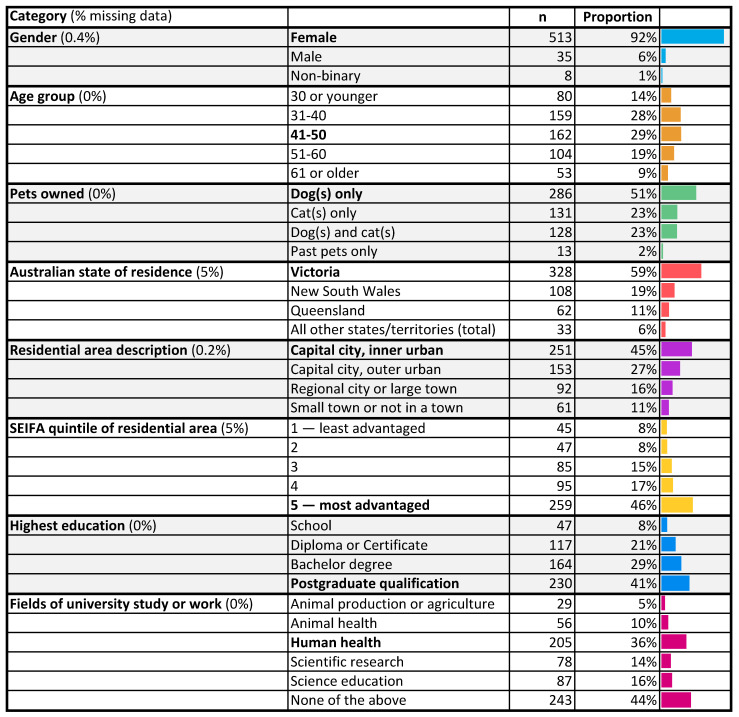
Demographics of the 558 included survey participants (excluding insufficiently complete surveys).

Participants were overwhelmingly female (92%). Most were aged 31–50 (58%), lived in the south-eastern Australian state of Victoria (59%), where the research team was based, and lived in the inner suburbs of a capital city (45%). A high proportion (41%) had a postgraduate educational qualification and 43% were in the highest quintile of socio-economic advantage (Figure 1).

More than half the participants cited a field of work or tertiary study that might have exposed them to the concept of AMR. Of particular note were the 44% of all participants who cited a background in human health and/or animal health. There was also considerable overlap between participants who cited a health background and those who had a postgraduate qualification; 51% (125/246) of postgraduate-qualified participants had a health background.

### 2.2. Pet Owner Expectations of the Veterinary Consultation, Treatment Preferences and Trust

Responses to these questions indicated that most owners viewed their veterinarians as trusted experts and perceived that there was significant value in a veterinary consultation, even when no medications were given. However, a significant number of pet owners indicated that they had expected and sometimes explicitly requested antibiotics from a veterinarian.

**Figure 2:**
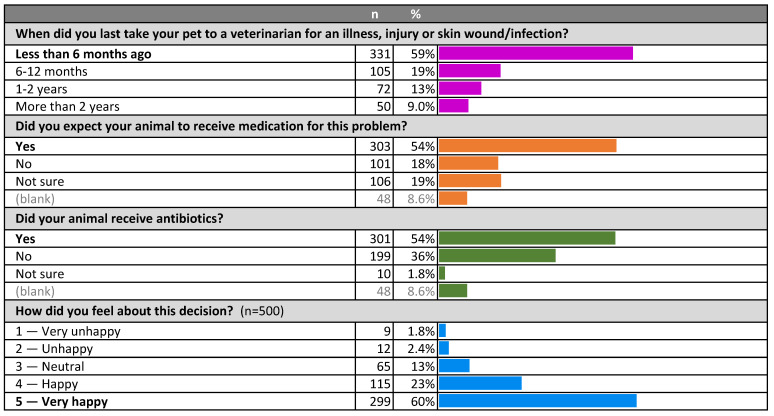
Recency of visit to veterinarian, expectations of therapy and satisfaction with treatment decision.

Reflecting on their most recent veterinary consultation for an illness, injury or skin infection or wound, most participants (54%) reported having had an expectation that their animal would receive some sort of medication, and 54% said their animal had received antibiotics (Figure 2). The vast majority (83%) were happy or very happy with the decision to use antibiotics, or not.

**Figure 3:**
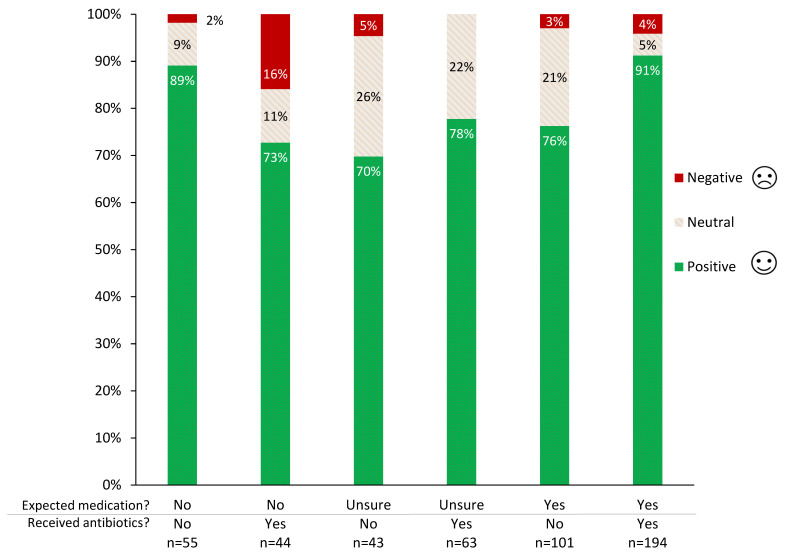
Owner feelings about treatment decision in their most recent veterinary consultation for animal illness or injury, by expectation of medication and receipt of antibiotics.

The subgroups with the highest positive evaluations were those for whom the consultation outcome aligned with their expectations; either receiving antibiotics when they had expected some medication (91% positive), or not receiving antibiotics when they had not expected medication (89% positive) (Figure 3).

However, it is noteworthy that even in these ‘aligned’ groups, there was a small minority who were still unhappy with the decision to give or withhold antibiotics. The subgroup with the highest proportion of negative evaluations was owners who had not expected medication, but received antibiotics (Figure 3). Among owners who had expected medication, not receiving antibiotics decreased the proportion of positive owner evaluations by 15% and increased the proportion of neutral owner evaluations by a similar amount, but had no apparent impact on negative evaluations (4% negative with antibiotics, 3% negative without). No significant differences were found between participant subgroups, including between those with a health background (HB) and those with no health background (NHB).

**Figure 4:**
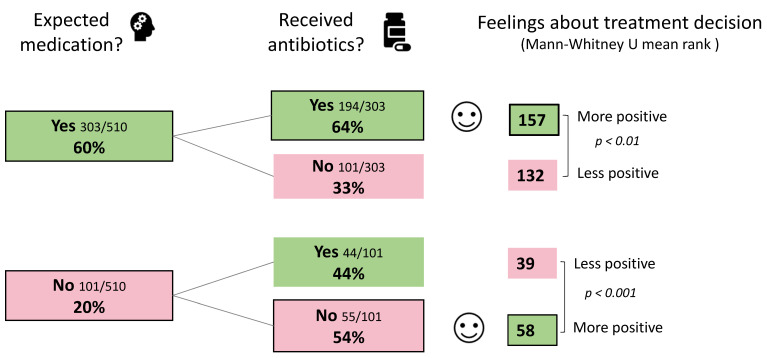
Owner feelings about treatment decision, based on their expectations about medication for their pet and whether they received antibiotics. ‘Unsure’ responses were removed, therefore totals are <100%.

Owners felt significantly more positive when their expectations about medication of their pet were aligned with the final decision about administering antibiotics [*p* < 0.01 for those expecting their pet to be medicated, *p* < 0.001 for those not expecting their pet to be medicated] (Figure 4).

**Figure 5:**
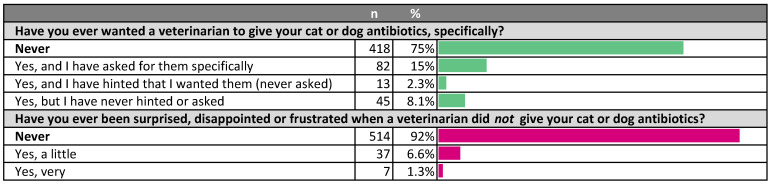
Owner desire, hints and requests for antibiotics for their pets.

Most owners (75%) said that they had never specifically wanted a veterinarian to give antibiotics to their pet Figure 5). This proportion was similar for HB (78%) and NHB owners (72%). Within the 25% of respondents who said that they had been surprised, disappointed or frustrated when a veterinarian had not given them antibiotics for their pet, 15% said they had specifically requested them, 2% said they had hinted but not asked specifically, and 8% said that they had not communicated their desire for prescription of antibiotics for their pet to the veterinarian. HB owners were almost twice as likely to have asked for antibiotics than NHB owners (OR 1.98, *p* < 0.01, 95% CI 1.2 to 3.2).

**Figure 6:**
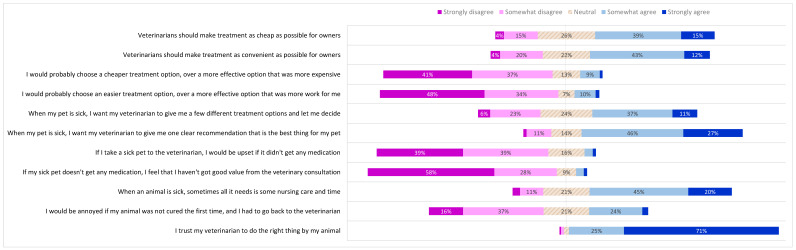
Pet owner expectations of the veterinary consultation, treatment preferences and trust. Pet owners were asked to what extent they agreed with each statement on the left. Proportions of responses to each statement corresponding to each of the five points of the Likert scale are shown. Data labels omitted where value is <4%.

**Figure 7:**
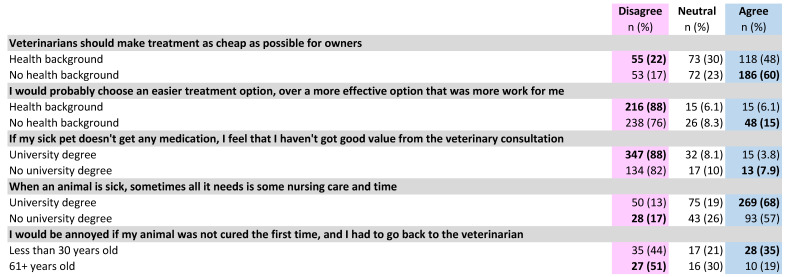
Differences by respondent demographic category in pet owner expectations of a veterinary consultation, treatment preferences and trust. Bold text indicates a significantly higher subgroup result (*p* < 0.05).

Although owners mostly agreed that veterinarians should make treatment as cheap (55%) and convenient (55%) as possible for pet owners (Figure 6), higher proportions indicated that they would *not* prioritize effectiveness over cheapness (77%) nor over convenience (81%) (Figure 6). NHB owners were more likely to feel that veterinarians should make treatment cheap (60%) than HB owners (48%) (difference 12%, *p* < 0.01, 95% CI 3.7 to 20%) (Figure 7). Notably, socio-economic advantage (SEIFA quintile) did *not* make a significant difference to this response [*p* = 0.20] HB owners were also significantly less likely (6.1%) than NHB owners (15%) to indicate that they would choose a more convenient treatment over a more effective treatment (difference 8%, *p* < 0.001, 95% CI 3.7 to 14%) (Figure 7).

More owners agreed (49%) than disagreed (29%) that they wanted to be given a few different treatment options. However, most pet owners (73%), including those who wanted multiple options, still wanted their veterinarian to recommend a single best option.

Very few pet owners indicated that they would feel upset if their sick pet did not receive any medication (5.2%) or that they had not obtained good value if they left the consultation without any medication (5.0%). Those without a university education were more likely (7.9%) than those with a university education (3.8%) to link consultation value with receiving medication (difference 4%, *p* < 0.05, 95% CI 1.4% to 8.1%) (Figure 7). Relatedly, most owners (65%) agreed that sometimes animals needed only nursing care and time to get better. Those with a university education were more likely to agree with this (68%) than those without (57%) (difference 12%, *p* < 0.01, 95% CI 2.8 to 20%) (Figure 7).

About one-quarter of owners (27%) indicated that they would be annoyed if their animal was not cured after the first veterinary consultation and needed to return to the clinic. Agreement with this statement was significantly higher (35%) in owners aged under 30, than in those aged 61 or older (19%) (difference 16%, *p* < 0.05, 95% CI 0.42 to 32%) (Figure 7).

The level of trust that pet owners had in their veterinarians was extremely high; 96% agreed they trusted their veterinarian to do the right thing by their pet, including 71% who strongly agreed with this statement.

### 2.3. Pet Owner Opinions on Antibiotic Use; Knowledge of Microbiology and Antibiotic Resistance

Most respondents were broadly aware of the risks and limitations of antibiotics, and, accordingly, indicated a willingness to avoid them if possible, but there was limited awareness of risks beyond the treated animal, even in HB owners.

**Figure 8:**
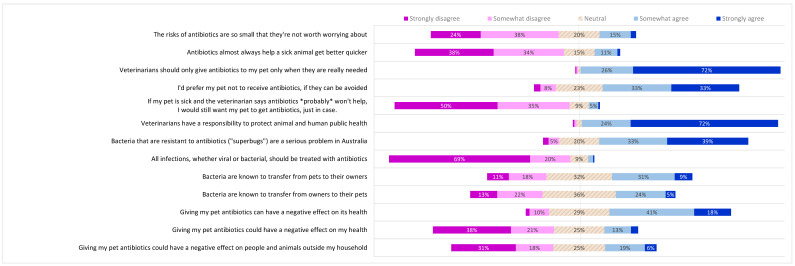
Pet owner opinions on antibiotic use; knowledge of microbiology and antibiotic resistance. Pet owners were asked to what extent they agreed with each statement on the left. Proportions of responses to each statement corresponding to each of the five points of the Likert scale are shown. Data labels omitted where value is <4%.

**Figure 9:**
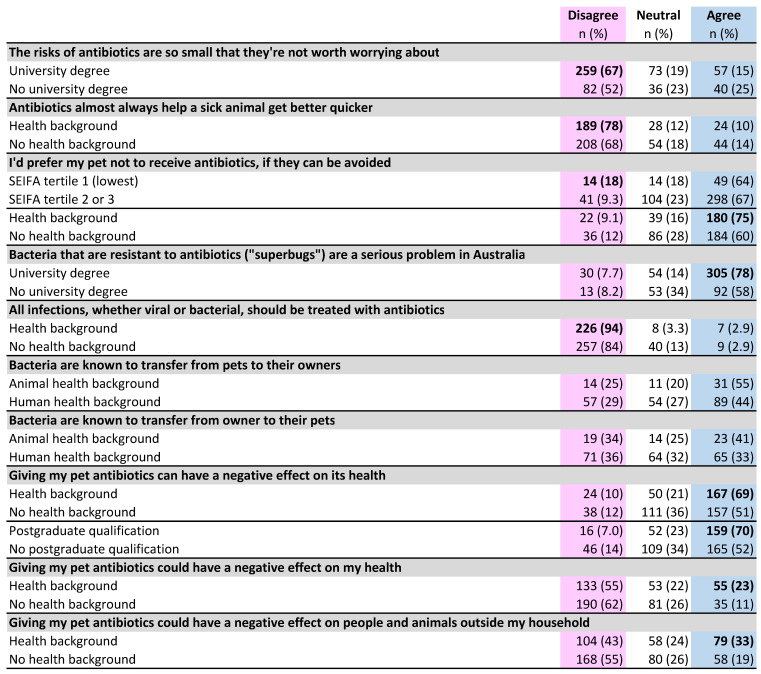
Differences by respondent demographic category in pet owner opinions for antibiotic use, knowledge of microbiology and antibiotic resistance. Bold type indicates a significantly higher subgroup result (*p* < 0.05).

A minority of pet owners (18%) agreed with the statement that the risks of antibiotics were negligible (Figure 8). Although the majority (62%) believed that there were significant risks (by disagreeing with the statement), there was a substantial difference between those with (67% disagree) and those without a university education (52% disagree) (difference 15%, *p* < 0.05, 95% CI 5.7 to 24%). Most (73%) also disagreed that antibiotics almost always make a sick animal better faster; a negative response was more common in HB owners (78%) than NHB owners (68%) (difference 10%, *p* < 0.01, 95% CI 2.9 to 18%) (Figure 9).

Pet owners felt very strongly that veterinarians should only give antibiotics to their pet when they were really needed (26% somewhat agree + 72% strongly agree = 97%). However, fewer agreed that they would prefer their pet not to receive antibiotics if they could be avoided (33% somewhat agree + 33% strongly agree = 67%) and 10% of all participants disagreed with this statement, with a higher proportion (18%) of those in the lowest socio-economic tertile disagreeing more than other tertiles combined (difference 8%, *p* < 0.05, 95% CI 1.5 to 16%). The group that most wanted to avoid antibiotics for their pet if possible (75% agree) were HB owners, compared with 60% of NHB owners (difference 15%, *p* < 0.001, 95% CI 6.6 to 23%) (Figure 9). Only 5% of pet owners indicated that they would want antibiotics for their pet when they probably would not help, ‘just in case’.

Pet owners were virtually unanimous (24% somewhat agree + 72% strongly agree = 96%) that veterinarians have a responsibility to protect both animal and human health.

Questions exploring pet owners’ understanding of microbiology and antibiotic resistance (Figure 8) received a significantly greater proportion of neutral responses than the questions about their opinions, expectations and preferences (25% for knowledge questions; 14% for other survey questions) (difference 11%, *p* < 0.001, 95% CI 10 to 13%). Most respondents (73%) agreed that antibiotic-resistant bacteria represented a serious problem in Australia, but agreement was much higher in those with a university degree (78%) than in those without a university education (59%) (difference 19%, *p* < 0.001, 95% CI 12 to 28%) (Figure 9).

The vast majority of respondents (89%) indicated an awareness of the therapeutic limitations of antibiotics, disagreeing with the statement that all bacterial and viral infections should be treated with antibiotics. Disagreement was significantly higher in HB owners (94%) than NHB owners (82%) (difference 9.8%, *p* < 0.001, 95% CI 4.4 to 15%).

The potential for transfer of bacteria between pets and their owners was not widely recognised, although a significantly higher proportion of respondents agreed that bacteria are known to move from pets to their owners (39%) than from owners to pets (29%) (difference 10%, *p* < 0.001, 95% CI 4.4 to 16%) (Figure 9). Of those who agreed with the first statement, only two-thirds agreed with the second statement (10% disagreed; 22% neutral).

HB owners were more likely than NHB owners to agree that bacteria are known to transfer from pets to their owners (46% compared to 34%) [difference 12%, *p* < 0.01, 95% CI 3.3 to 20%], and from owners to their pets (34% compared to 25%) (difference 8.8%, *p* < 0.05, 95% CI 3.2 to 18%). Respondents with an animal health background were more likely to agree with each of these statements than those with a human health background (41% compared to 33%, and 55% compared to 44%, respectively), but these differences were not statistically significant (Figure 9). Post-hoc analysis showed that a sample size several times larger than this one would be required for this difference to reach significance.

Awareness of adverse effects of antibiotics on the individual animal was high (60%), but only 16% agreed that there was a potential risk to themselves when their pet had antibiotics. Again, a health background was associated with increased awareness of direct adverse effects on pet’s health (69% compared to 51%) (difference 18%, *p* < 0.001, 95% CI 9.7 to 26%) as were postgraduate qualifications (70% compared to 52%) (difference 18%, *p* < 0.001, 95% CI 10 to 27%). HB owners were also more aware than NHB owners of the potential for antibiotics given to the pet to have negative effects on their own health (23% compared to 11%) (difference 11%, *p* < 0.001, 95% CI 5.1 to 18%).

Almost half (49%) of the respondents did not agree that antibiotics in their pet could pose a risk to animals and humans outside their household and only 25% agreed that this would pose a wider risk to animals and people outside their own household. HB owners were significantly more likely (33%) to recognise the broader risks of antibiotic use, compared with NHB owners (19%) (difference 14%, *p* < 0.001, 95% CI 6.5 to 21%) (Figure 9).

### 2.4. Communication Preferences

#### 2.4.1. Communication Preferences When Not Prescribing Antibiotics, and Delayed Prescribing Preferences

When a veterinarian declines to give antibiotics to an unwell pet or delays prescribing of antibiotics, owners indicated that reassurance was important, but different subgroups of owners expressed different preferences for how such reassurance was framed.

**Figure 10:**
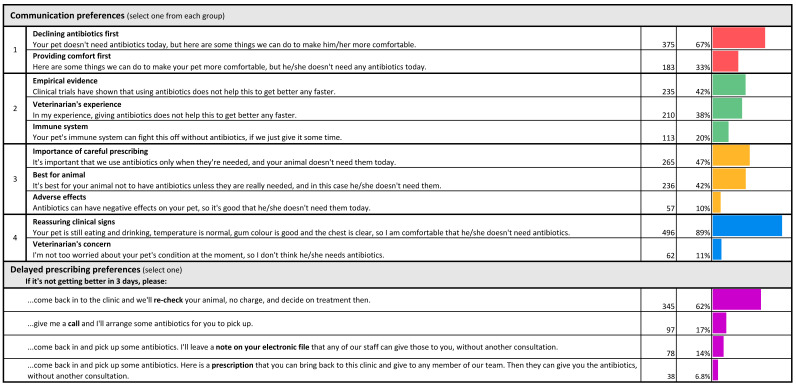
Communication preferences when not prescribing antibiotics, and delayed prescribing preferences.

Participants were shown four sets of options about communication preferences and one set of options about delayed prescribing (Figure 10).

Most pet owners (67%) preferred to be told that their animal did not need antibiotics before being told about ways their pet can be made more comfortable; only 33% preferred to be told about ways their pet can be made more comfortable before being told their animal did not need antibiotics.

From a selection of three reasons for why antibiotics were not needed, highlighting the role of the animal’s immune system in controlling infection, the veterinarian’s past experience and data from clinical trials, participants indicated that data on clinical trials were the preferred reason for not using antibiotics (42%). However, this varied with the respondent’s level of education and background in human or animal health. Those whose highest level of education was completion of high school were twice as likely to prefer the veterinarian’s experience (47%) over clinical trial data (23%). For HB owners and for those with any postgraduate qualification, this was reversed: 54% preferred to hear about clinical trial data and only 30% preferred to hear about the veterinarian’s experience. Only 20% of all participants selected the argument that their animal’s immune system would resolve the infection.

Fairly equal proportions of respondents preferred the explanations that veterinarians should only use antibiotics when they are needed (47%) and that avoiding antibiotics is best for the pet (42%) as reasons for not treating with antibiotics. The explanation that antibiotics can have adverse effects was the least preferred of these three options (10%).

Given a choice between being reassured that their pet’s clinical signs did not indicate a need for antibiotic therapy and the veterinarian’s low level of concern about their pet’s condition, most respondents (89%) preferred reassurance based on the animal’s clinical signs. Participants with the lowest level of education (school only) were more likely than all other groups (23%) to select the veterinarian’s low level of concern as a basis for not initiating antibiotic therapy, compared with just 11% of the participants with a postgraduate education (difference 13%, *p* < 0.05, 95% CI 2.0 to 23%).

With regard to delayed prescribing, the most preferred approach (62%) was to have the animal re-checked at the clinic if needed, without charge and without promising antibiotics. The least preferred option (6.8%) was receiving a paper prescription for antibiotics to present at the clinic if needed.

#### 2.4.2. Reasons to Give a Course of Antibiotic Tablets, Rather Than a Single, Long-Acting Injection

**Figure 11:**
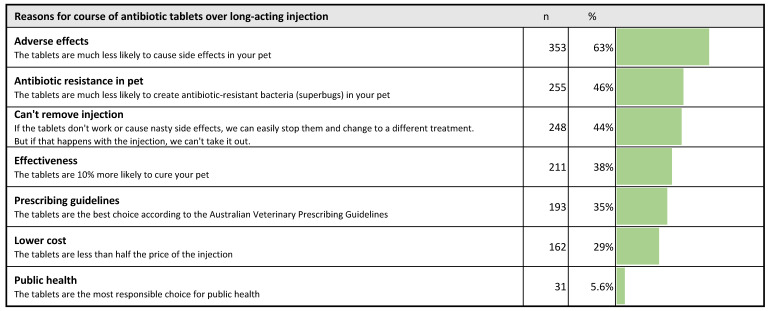
Pet owner preferences for reasons to give a course of antibiotic tablets rather than a single, long-acting antibiotic injection. Respondents could select up to three statements that they found convincing, so the total is >100%.

Pet owners indicated that the risk of adverse effects and antibiotic resistance in their pet would be the reasons most likely to motivate them to select a course of antibiotic tablets (twice daily for 5 days) for their pet, over a single, long-acting antibiotic injection (Figure 11).

**Figure 12:**
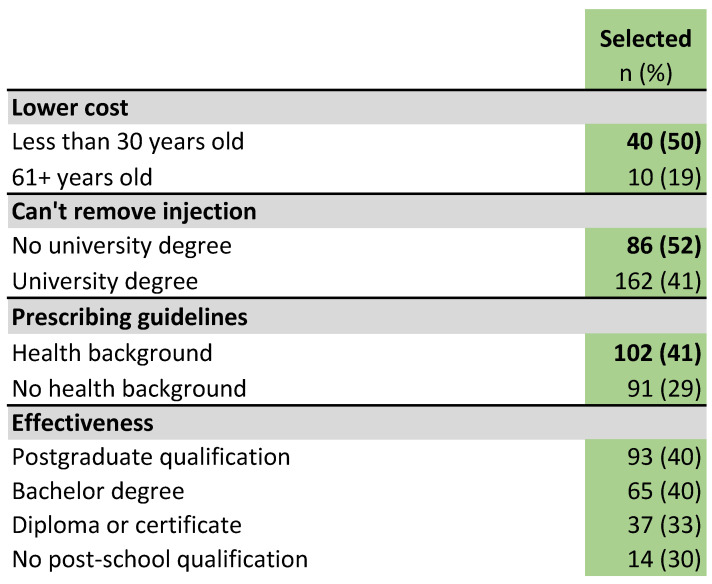
Differences by respondent demographic category in popularity of reasons to give a course of antibiotic tablets instead of a long-acting antibiotic injection. Bold type indicates a significantly higher subgroup result (*p* < 0.05).

The most popular response (selected by 63%) was a lower likelihood of adverse effects in their pet, followed by a decreased risk of antibiotic-resistant bacteria in their pet (46%). By far the least popular reason (6%) was that the tablets were the most responsible choice for public health. The lower cost of tablets was also a relatively unpopular reason (29%). Notably, there was no difference in the popularity of a lower cost between different quintiles of socio-economic advantage (based on residential postcode), but it was significantly more popular among those aged 30 or younger (50%) than in those aged 61 or older (19%) (difference 31%, *p* < 0.01, 95% CI 12 to 50%) (Figure 12).

The argument that an injected long-acting antibiotic could not be removed if it did not work or was to cause adverse effects was selected by 44% of all participants, but was more popular among owners who had no university education (52%) than among those who had a university qualification (41%) (difference 11%, *p* < 0.05, 95% CI 1.9 to 20%) (Figure 12).

Just over a third of all respondents (35%) favored the argument that tablets were the option recommended by veterinary prescribing guidelines. In HB owners, this proportion was 41%, while for NHB owners it was 29% (difference 12%, *p* < 0.01, 95% CI 4.3 to 20%). The argument that the course of tablets had increased effectiveness was more popular with participants with higher levels of education; 30% of those who had no post-secondary school qualifications chose this reason, increasing to 40% of those with a postgraduate qualification, but this difference was not statistically significant (Figure 12).

## 3. Discussion

Our results indicate that pet owners often expect and sometimes hint at, or explicitly request, antibiotics for their sick pets, but appreciate that there are risks to their pet from antibiotic use. Pet owners expressed a great deal of trust in their veterinarians, and general satisfaction with the outcome of a consultation, even when no medication is given. Satisfaction with a consultation in which medication is not provided may be increased if the veterinarian provides reassurance and the option for re-examination.

### 3.1. Pet Owner Expectations of the Veterinary Consultation, Treatment Preferences and Trust

Although a majority of pet owners felt that veterinarians should make treatment as cheap and convenient as possible for owners, a larger majority denied that when choosing between two treatment options, they would prioritize cheapness or convenience of treatment over effectiveness. This would suggest that owners care most about resolving their animal’s problem. Veterinarians should therefore emphasize to owners that judicious treatment, whether using a non-antibiotic therapy, or using an antibiotic with a lower importance rating, is at least as effective as more convenient options they might request.

The relatively low priority owners placed on cost is seemingly at odds with the findings of a recent survey of dog owners from North America [20], which found that low cost of treatment was more important (accounting for 47% of dog owner preferences) than ease (31% of owner preferences). However, the strong signal that convenience is less important to owners than effectiveness is also reflected in a cat owner survey in the UK, in which 88% of owners indicated that getting the most appropriate antibiotic was more important than ease of administration [21]. These findings are particularly encouraging for AMS interventions aimed at reducing use of high importance (but rarely the most appropriate) antibiotics, such as cefovecin and enrofloxacin.

Owners in this study indicated that the value they perceived in the veterinary consultation was not necessarily attached to the receipt of medication; however, more than half of surveyed pet owners (54%) had expected some sort of medication at their most recent presentation of an unwell animal. A similar proportion of parents (50%) expected antibiotics when they presented their children to a general practice doctor in a USA study, but only 1% of parents had made an explicit request [24]. Although the proportion of pet owners who specifically expected antibiotics at their most recent consultation cannot be determined from our survey, a quarter said they had, at some stage in the past, been surprised, disappointed or frustrated when a veterinarian had not provided antibiotics for their pet, and 15% had specifically asked for antibiotics. This indicates substantial public demand for antibiotics for pets, and is supported by qualitative studies of companion animal veterinarians in the UK [14,25] and The Netherlands [12]. Several studies of doctors have found that implicit and explicit requests for antibiotics, and even the client simply questioning the treatment plan, increase the likelihood of prescription of antibiotics [26,27,28] 

Unsurprisingly, pet owners were happiest when their pre-consultation expectations of medications for their pet aligned with receiving antibiotics, or not. However, it is perhaps somewhat surprising that the highest proportion of unhappy respondents were those whose animals had received antibiotics when the owner had not expected any medication, rather than those who expected medication and were not provided antibiotics for their pet. One possible explanation is that many respondents who expected their pet to receive medication in fact received non-antibiotic medications, which satisfied the owners. Another possibility is that those who had not expected medications for their pet felt that the antibiotics that they were given did not adequately address their animal’s problem. Qualitative research with pet owners should be helpful in clarifying this.

Notably, respondents indicated a very high degree of trust in their veterinarians to do the right thing by their pet, a finding that is reflected in qualitative studies of pet owners [22,23]. This degree of trust is helpful in facilitating conversations between veterinarians and pet owners about responsible antibiotic use.

### 3.2. Pet Owner Opinions on Antibiotic Use; Knowledge of Microbiology and Antibiotic Resistance

As expected, respondents with higher levels of education and those with health backgrounds had higher levels of knowledge about microbiology and antibiotic resistance and their opinions on antibiotic use aligned more closely with judicious prescribing principles.

Across all demographic groups, cat and dog owners appeared to understand that antibiotics are not indicated for viral infections, with a large majority disagreeing that all infections (viral and bacterial) should be treated with antibiotics, suggesting that this message has been successfully conveyed to the Australian public, including through initiatives such as ‘Not All Bugs Need Drugs’ [29]. This is similar to the findings of a recent study of cat owners in the UK [21].

Prescribing antibiotics ‘just in case’ was endorsed by only one in twenty of our participants, contrasting with interviews with pet owners in the US, where most participants said that they would like their pet to receive antibiotics even when there was no clear benefit [23]. It is uncertain whether these contrasts are a result of the methodological differences between the studies, true differences in the attitudes of the sample populations, or both.

However, One Health concepts were generally poorly understood. A minority of all pet owners appreciated that bacteria move from pets to their owners and a smaller minority knew that bacteria move from owners to pets. It is somewhat surprising that there was higher awareness of animal-to-human bacterial transfer than human-to-animal transfer, and even more surprising that this difference existed even among participants with animal and human health backgrounds. Similarly, only a small minority of pet owners recognised that giving antibiotics to their pet could have an adverse effect on them, on other animals in the household, and on animals and humans outside the household. This finding is supported by interviews with US pet owners [23], none of whom were concerned that the same antimicrobials used in their pets are also used in humans; indeed some actually found it comforting. Therefore, AMS messages for the general public, whether in a veterinary or human medical setting, should not assume an understanding of One Health and the potential for bi-directional inter-species transmission of AMR.

Interestingly, 43% of cat owners in the UK agreed that using antibiotics in animals can reduce the effectiveness of antibiotics in humans, implying an understanding of inter-species transfer of resistant bacteria. However, interviews with pet owners in the UK suggested that most pet owners tend to attribute this effect to antibiotic use in food-producing animals (and human consumption of those products) [14]. Additionally, we know that many people conceptualize AMR as a property of the treated human or animal body, similar to the tolerance induced by opioid use, rather than a property of resident bacteria [15,23], and that many lay people equate the presence of bacteria with infection [30]. It follows that even pet owners who understand AMR as a property of bacteria, but who do not know that their pet is colonized by trillions of bacteria in the absence of infection, will struggle to understand the off-target and long-term effects of antibiotic use. Interviews with veterinarians and pet owners have also highlighted significant gaps in pet owners’ understanding of how AMR develops and spreads [14,23].

**Figure 13:**
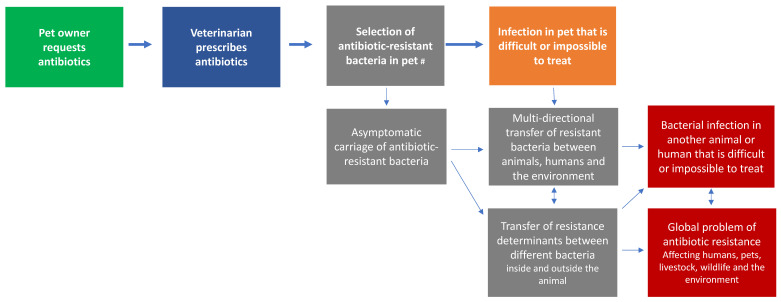
Owner behaviour (green) and public health outcomes (red) are linked by microbiological concepts (grey) that are poorly understood. The more direct potential adverse outcome for the pet (orange) is simpler to communicate, and remains logical even if the pet owner holds the ^#^ common misconception that antibiotic resistance is a property of the animal’s body.

Leveraging the public’s concern about antibiotic resistance (as a broad concept) to drive change in their behaviour around use of antibiotics in pets would therefore first require pet owners to link their behaviour with antibiotic resistance at a broader level. However, the microbiological concepts that link pet owner behaviors and the ‘big picture’ outcomes of optimal antibiotic stewardship are, in fact, quite complex (Figure 13) and not commonly understood, even by our respondents with health backgrounds and high levels of education. As it is difficult to communicate these concepts concisely, public health arguments against unnecessary antibiotics are not well suited to waiting-room posters and 10-min veterinary consultations. It is likely to be more effective to use messages about the potential for antibiotic-resistant infections in their pet, where the link is far more direct and is not hampered by common misunderstandings. However, individual owners who have very high health literacy, or owners in countries such as Sweden, where the population generally has high health literacy [31], may respond to messages about the public health benefits of avoiding unnecessary antibiotic use.

### 3.3. Communication Preferences

To our knowledge, this is the first quantitative study eliciting the preferences of pet owners for the way veterinarians communicate with them about antibiotics. In the most closely analogous setting of a parent and child consulting a doctor, an in-depth study of 570 consultations [32] found that, when antibiotics were not prescribed, parents were more satisfied when the doctor first explained what could be done to make the child feel better, before explaining that no antibiotics would be prescribed. We thus hypothesized that pet owners were likely to want to hear, *‘Here are some things we can do to make your pet more comfortable,’* before being told their pet would not be prescribed antibiotics. In fact, the opposite was true, with two-thirds of owners preferring to first hear the message, ‘Your pet doesn’t need antibiotics today’. However, as this was always the first of the questionnaire responses presented, order bias towards the first message on the list [33] may have contributed to the apparent preference for this message.

Different groups of respondents placed different importance on empirical data (‘Clinical trials have shown…’ and ‘Your pet is still eating and drinking…’) compared with the veterinarian’s experience or personal concern (‘In my experience, giving antibiotics doesn’t help…’ and ‘I’m not too worried about…’). Clients with lower levels of education clearly preferred messages about the veterinarian’s experience or personal concern, whereas clients with higher levels of education preferred messages invoking empirical data. This suggests that when declining to give antibiotics, veterinarians, and indeed doctors, may have more success if they tailor their communication to the person’s level of education, or, alternatively, they could incorporate both sets of messages when declining to provide antibiotics.

Delayed prescribing is an effective AMS strategy that is used commonly in both veterinary and medical consultations, when antibiotics are probably or definitely not indicated, and has been found to result in improved client satisfaction when no antibiotics are provided [24]. In a veterinary setting, the veterinarian would typically ask the owner to monitor the animal at home for particular changes, that would trigger either a re-examination or the beginning of a course of antibiotics. In this study, pet owners overwhelmingly preferred to come back into the clinic for a recheck, at no charge, to simply collecting antibiotics without a re-check. This suggests that many owners are more interested in the reassurance provided by the re-check, than in receiving antibiotics specifically. However, it is unlikely to be financially sustainable for veterinary clinics to provide free re-checks on a routine basis. In future qualitative studies about pet owner attitudes to delayed prescribing, it would be worth exploring owner preferences for a rec-heck, if it attracted a consultation fee.

Apart from discussions with some pet owners when declining antibiotics, companion animal veterinarians also report challenging conversations with some pet owners, especially cat owners-who prefer a long-acting injectable antibiotic, cefovecin, over oral antibiotic courses. Although undeniably convenient, cefovecin is classified by the World Health Organization as a Highest Priority Critically Important Antimicrobial (HPCIA), and should not be used empirically. In addition, its 14-day duration of action is longer than the duration of treatment recommended in small animal prescribing guidelines for common indications for antimicrobial treatment [34,35]. Veterinarians therefore need effective ways to decline cefovecin and instead persuade pet owners to give alternative treatment (often tablets of a lower-importance antibiotic), while maintaining owner satisfaction. Our results show that owners prefer arguments centered on the health of their pet, the possibility of their pet experiencing direct adverse effects from the antibiotic and the possibility of ‘superbugs’ developing in their pet, and effectiveness over arguments around relative cost, public health or veterinary prescribing guidelines. Arguing for an antimicrobial because it is of lower importance to human and animal medicine is also unlikely to be helpful; most dog owners in the North American study actually indicated a preference for their dog to receive high-importance antimicrobials over lower-importance antimicrobials [20]. Instead, framing a lower-importance antibiotic as a safer and more effective choice for their pet is likely to be more compelling.

Interestingly, the very strong positive responses (96%) to the statement ‘Veterinarians have a responsibility to protect animal and human public health’ (see Pet owner opinions) are in stark contrast to the lack of popularity (5.6%) of the public health argument that a veterinarian could give for using the more responsible antibiotic tablets over the less responsible long-acting injection. It is possible that many pet owners simply do not see a link between their veterinarian’s responsibility to public health (a broad concept that they might not associate with specific actions) and decisions about their pet, which are salient, personal and emotional. Alternatively, pet owners might feel that their veterinarian should execute their responsibility to public health when treating other animals, but not theirs. Again, qualitative research would be helpful to confirm and understand this apparent disagreement.

### 3.4. Strengths and Limitations

Three quarters (74%) of respondents reported owning a dog and half (49%) had a cat; these proportions broadly mirror the relative popularity of these animals across Australian households [1]. Survey respondents were overwhelmingly female (92%), similar to a UK cat owner survey (91%) [21], but approximately two-thirds of pet owners in a previous national survey were found to be women [1], and in our experience, an even higher proportion of the owners who present pets to veterinary clinics are women. The heavily urban distribution of responses is also largely reflective of the national population, 90% of whom reside in urban areas [36]. A more even spread of responses from different states of Australia would have been preferable. A very high proportion of respondents (70%) had tertiary qualifications, compared with a national average of 28% of those 15–64 years old, and a high proportion (43%) had postgraduate qualifications (the national average for those aged 15–64 years is 8.8%) [37]. Additionally, a disproportionately high proportion of respondents (44%) were tertiary educated in and/or worked in health fields, compared with an estimated 10% of the national population [37]. Almost half of our respondents (46%) were in the socio-economic quintile of highest advantage, based on their postcode. This selection bias was anticipated, and is likely to have been in part a consequence of the social networks through which the survey was promoted. However, systematic subgroup analyses allowed us to distinguish the effects of this bias.

Respondents’ recall of past veterinary interactions, and their prediction of their response to specific situations, may be imperfect, as people commonly idealize and oversimplify their mental representation of scenarios, ignoring factors that are salient in reality [38]. For example, owners who indicate that convenience of treatment is not as important as effectiveness may be less likely to feel that way if their cat bites them while they are trying to administer tablets, and owners who indicate that cost is not important to them could well feel differently if their financial circumstances change. Nevertheless, knowing how owners would like to behave provides important insights into their idealized priorities and allows us to design effective communication strategies for AMS.

## 4. Materials and Methods

Dog and cat owners were recruited through a combination of social media posts (mainly in cat and dog interest groups), posters with QR codes in veterinary clinic waiting rooms and through the authors’ personal contacts. Recruitment materials invited pet owners who had taken their pet to a veterinarian for an illness or injury in the previous two years to participate. Veterinarians were not specifically excluded from participating, but because many practicing veterinarians treat their own pets, the stipulation that the survey should be undertaken by those who had taken their pet to a veterinarian made it unlikely that veterinarians themselves would participate.

Participants undertook an anonymous online survey, using Qualtrics (Qualtrics, Provo, UT, USA), from February to April 2021. The survey asked a range of demographic questions, including gender, age, residential postcode and highest level of education. These data were deliberately sought in anticipation of the selection bias commonly seen in health studies, where older females with higher levels of education and higher socio-economic advantage are overrepresented [39,40,41]. Participants were also asked whether they had received university education or worked in scientific or health fields, as disproportionate responses were expected from these groups, and they could reasonably be expected to have a greater interest than the general population in antimicrobial use in animals.

The demographic questions were followed by questions about recent interactions with veterinary care, attitudes and knowledge about veterinary treatment and their preferences with respect to communication from veterinarians (Questionnaire: Supplementary Materials, Document S1). Most of the questions were answered with a 5-point Likert scale, ranging from strongly agree to strongly disagree; questions on veterinary communication preferences asked the participant to select the most preferred message(s). There were two free-text questions that enabled the participant to provide context for their other responses. In some cases, owners provided their pet’s diagnosis and outcome, or described the difficulty they experienced administering oral antimicrobials to their pet.

A question about owners’ preferred reason for using a course of antibiotic tablets, rather than a single, long-acting antibiotic injection, was included in the survey (Q22) because veterinarians have reported having this conversation with pet owners who request a single injection of cefovecin, when multiple doses of an oral antibiotic would be a more appropriate choice.

In this survey of the general public, the term ‘antibiotics’ is used as it is in common parlance in the English-speaking world, to describe all antimicrobial medications directed at bacteria, including those not strictly classed as antibiotics. We have used it throughout the rest of this paper to mean all these medications. In the pet owner survey, the term ‘vet’ was used to mean veterinarian, as this is the most commonly used term used by the local population; however, for international readability we have replaced it with the term ‘veterinarian’ throughout this manuscript.

Data were cleaned and analysed using Microsoft Excel and IBM SPSS. Differences in medians (the level of Likert scale agreement) between two groups were analysed using Mann-Whitney U tests, retaining all five levels of response. To examine proportions of responses across different participant groups, responses were collapsed into a 3-point Likert response, with ‘strongly agree’ and ‘somewhat agree’ combined, and ‘somewhat disagree’ and ‘strongly disagree’ also combined. Differences in proportions were analysed using a two-tailed z-test, with a significance level of 0.05. Pearson’s chi-squared test was used to test the effect of participants’ socio-economic advantage on their responses.

The socio-economic advantage of participants was estimated using their residential postcode, and the corresponding Socio-Economic Index for Area (SEIFA) centile for that postcode, calculated by the Australian Bureau of Statistics using data from the most recent national Census [42].

## 5. Conclusions

This survey has provided the first data on pet owners’ communication preferences when a veterinarian is declining to give antibiotics, using a delayed prescribing strategy, or encouraging a more appropriate (but less convenient) treatment. This study also provides new data on Australian pet owners’ opinions, preferences and knowledge about antibiotics.

There is significant owner desire for antibiotics for unwell pets, but most owners also recognise antibiotic resistance as a serious problem, and are rarely disappointed when antibiotics are not given. Owners place a higher priority on their pet receiving the most effective treatment than on treatment being cheap or convenient. When declining to provide antibiotics, veterinarians would do well to detail the animal’s reassuring clinical signs, mention supporting clinical trial data (where available) and speak of their experiences when the condition has resolved without antibiotics. If pressed, the prescriber could mention the importance of using antibiotics only when needed, and the direct risks of adverse effects and encouraging ‘superbugs’ in that particular animal. However, unless there is very high health literacy, or ample consultation time, it is probably wise to avoid invoking the broader risks of antibiotic use, as public understanding of normal microbiomes and transfer of bacteria between animals and humans is limited.

These findings can be applied by veterinarians, and indeed other antibiotic prescribers, including human medical practitioners, particularly those treating young children presented by their parents, in order to have more effective conversations about antibiotics with patients, parents and pet owners. Insights from this study should also be considered when devising public AMR education campaigns.

## Data Availability

The data presented in this study are available in Supplementary Material.

## Supplementary Materials

The following are available online at https://www.mdpi.com/article/10.3390/antibiotics10111326/s1, Document S1: Pet owner questionnaire; Figure S1: Demographics of respondents excluded from analysis

## References

[1] Animal Medicines Australia (2019). Pets in Australia: A National Survey of Pets and People. https://animalmedicinesaustralia.org.au/report/pets-in-australia-a-national-survey-of-pets-and-people/.

[2] Hardefeldt L.Y., Selinger J., Stevenson M.A., Gilkerson J.R., Crabb H., Billman-Jacobe H., Thursky K., Bailey K.E., Awad M., Browning G.F. (2018). Population wide assessment of antimicrobial use in dogs and cats using a novel data source—A cohort study using pet insurance data. Vet. Microbiol..

[3] Buckland E.L., O’Neill D., Summers J., Mateus A., Church D., Redmond L., Brodbelt D. (2016). Characterisation of antimicrobial usage in cats and dogs attending UK primary care companion animal veterinary practices. Vet. Rec..

[4] Singleton D.A., Sánchez-Vizcaíno F., Dawson S., Jones P.H., Noble P.J.M., Pinchbeck G.L., Williams N.J., Radford A.D. (2017). Patterns of antimicrobial agent prescription in a sentinel population of canine and feline veterinary practices in the United Kingdom. Vet. J..

[5] Schmitt K., Lehner C., Schuller S., Schüpbach-Regula G., Mevissen M., Peter R., Müntener C.R., Naegeli H., Willi B. (2019). Antimicrobial use for selected diseases in cats in Switzerland. BMC Vet. Res..

[6] Joosten P., Ceccarelli D., Odent E., Sarrazin S., Graveland H., van Gompel L., Battisti A., Caprioli A., Franco A., Wagenaar J.A. (2020). Antimicrobial Usage and Resistance in Companion Animals: A Cross-Sectional Study in Three European Countries. Antibiotics.

[7] Galarce N., Arriagada G., Sánchez F., Venegas V., Cornejo J., Lapierre L. (2021). Antimicrobial Use in Companion Animals: Assessing Veterinarians’ Prescription Patterns through the First National Survey in Chile. Animals.

[8] Goggs R., Menard J.M., Altier C., Cummings K.J., Jacob M.E., Lalonde-Paul D.F., Papich M.G., Norman K.N., Fajt V.R., Scott H.M. (2021). Patterns of antimicrobial drug use in veterinary primary care and specialty practice: A 6-year multi-institution study. J. Vet. Intern. Med..

[9] Hur B.A., Hardefeldt L.Y., Verspoor K.M., Baldwin T., Gilkerson J.R. (2020). Describing the antimicrobial usage patterns of companion animal veterinary practices; free text analysis of more than 4.4 million consultation records. PLoS ONE.

[10] De Briyne N., Atkinson J., Pokludov L., Borriello S.P., Price S. (2013). Factors influencing antibiotic prescribing habits and use of sensitivity testing amongst veterinarians in Europe. Vet. Rec..

[11] Currie K., King C., Nuttall T., Smith M., Flowers P. (2018). Expert consensus regarding drivers of antimicrobial stewardship in companion animal veterinary practice: A Delphi study. Vet. Rec..

[12] Hopman N.E.M., Hulscher M.E.J.L., Graveland H., Speksnijder D.C., Wagenaar J.A., Broens E.M. (2018). Factors influencing antimicrobial prescribing by Dutch companion animal veterinarians: A qualitative study. Prev. Vet. Med..

[13] King C., Smith M., Currie K., Dickson A., Smith F., Davis M., Flowers P. (2018). Exploring the behavioural drivers of veterinary surgeon antibiotic prescribing: A qualitative study of companion animal veterinary surgeons in the UK. BMC Vet. Res..

[14] Smith M., King C., Davis M., Dickson A., Park J., Smith F., Currie K., Flowers P. (2018). Pet owner and vet interactions: Exploring the drivers of AMR. Antimicrob. Resist. Infect. Control.

[15] Dickson A., Smith M., Smith F., Park J., King C., Currie K., Langdridge D., Davis M., Flowers P. (2019). Understanding the relationship between pet owners and their companion animals as a key context for antimicrobial resistance-related behaviours: An interpretative phenomenological analysis. Health Psychol. Behav. Med..

[16] Zoetis Australia Pty Ltd Convenia (Cefovecin Sodium), Product Information. https://websvr.infopest.com.au/LabelRouter?LabelType=L&Mode=1&ProductCode=60461.

[17] Burke S., Black V., Sánchez-Vizcaíno F., Radford A., Hibbert A., Tasker S. (2017). Use of cefovecin in a UK population of cats attending first-opinion practices as recorded in electronic health records. J. Feline Med. Surg..

[18] Hardefeldt L., Hur B., Verspoor K., Baldwin T., Bailey K.E., Scarborough R., Richards S., Billman-Jacobe H., Browning G.F., Gilkerson J. (2020). Use of cefovecin in dogs and cats attending first-opinion veterinary practices in Australia. Vet. Rec..

[19] Tompson A.C., Chandler C.I.R., Mateus A.L.P., O’Neill D.G., Chang Y.-M., Brodbelt D.C. (2020). What drives antimicrobial prescribing for companion animals? A mixed-methods study of UK veterinary clinics. Prev. Vet. Med..

[20] Stein M.R., Evason M.D., Stull J.W., McClure J.T., Weese J.S. (2021). Knowledge, attitudes and influencers of North American dog-owners surrounding antimicrobials and antimicrobial stewardship. J. Small Anim. Pract..

[21] Stallwood J., Shirlow A., Hibbert A. (2020). A UK-based survey of cat owners’ perceptions and experiences of antibiotic usage. J. Feline Med. Surg..

[22] Rhys-Davies L., Ogden J. (2020). Vets’ and Pet Owners’ Views About Antibiotics for Companion Animals and the Use of Phages as an Alternative. Front. Vet. Sci..

[23] Redding L.E., Cole S.D. (2019). Pet owners’ knowledge of and attitudes toward the judicious use of antimicrobials for companion animals. J. Am. Vet. Med. Assoc..

[24] Mangione-Smith R., McGlynn E.A., Elliott M.N., McDonald L., Franz C.E., Kravitz R.L. (2001). Parent Expectations for Antibiotics, Physician-Parent Communication, and Satisfaction. Arch. Pediatr. Adolesc. Med..

[25] Mateus A.L.P., Brodbelt D.C., Barber N., Stärk K.D.C. (2014). Qualitative study of factors associated with antimicrobial usage in seven small animal veterinary practices in the UK. Prev. Vet. Med..

[26] Mangione-Smith R., McGlynn E.A., Elliott M.N., Krogstad P., Brook R.H. (1999). The Relationship Between Perceived Parental Expectations and Pediatrician Antimicrobial Prescribing Behavior. Pediatrics.

[27] Mangione-Smith R., Elliott M.N., Stivers T., McDonald L.L., Heritage J. (2006). Ruling Out the Need for Antibiotics. Arch. Pediatr. Adolesc. Med..

[28] Ong S., Nakase J., Moran G.J., Karras D.J., Kuehnert M.J., Talan D.A. (2007). Antibiotic Use for Emergency Department Patients With Upper Respiratory Infections: Prescribing Practices, Patient Expectations, and Patient Satisfaction. Ann. Emerg. Med..

[29] Agriculture Victoria Not All Bugs Need Drugs. https://agriculture.vic.gov.au/livestock-and-animals/livestock-health-and-welfare/antibiotic-resistant-infections/information-for-vets.

[30] Galli I., Fasanelli R. (2020). Public understanding of science and common sense: Social representations of the human microbiome among the expert and non-expert public. Health Psychol..

[31] Vallin M., Polyzoi M., Marrone G., Rosales-Klintz S., Wisell K.T., Lundborg C.S. (2016). Knowledge and Attitudes towards Antibiotic Use and Resistance—A Latent Class Analysis of a Swedish Population-Based Sample. PLoS ONE.

[32] Stivers T. (2005). Non-antibiotic treatment recommendations: Delivery formats and implications for parent resistance. Soc. Sci. Med..

[33] Feenberg D., Ganguli I., Gaulé P., Gruber J. (2017). It’s Good to Be First: Order Bias in Reading and Citing NBER Working Papers. Rev. Econ. Stat..

[34] University of Melbourne (2019). Australian Veterinary Prescribing Guidelines, Dogs and Cats. https://vetantibiotics.fvas.unimelb.edu.au/about/resources/.

[35] Jessen L.R., Damborg P.P., Spohr A., Sørensen M., Langhorn R., Goericke-Pesch S.K., Houser G., Willesen J., Schjærff M., Eriksen T. (2019). Antibiotic Use Guidelines for Companion Animal Practice.

[36] Australian Bureau of Statistics (2019). Historical Population Data, 2016. https://www.abs.gov.au/statistics/people/population/historical-population/latest-release#data-download.

[37] Australian Bureau of Statistics (2020). Qualifications and Work 2018-19. https://www.abs.gov.au/statistics/people/education/qualifications-and-work/2018-19#data-download.

[38] Poon C., Koehler D., Buehler R. (2014). On the psychology of self-prediction: Consideration of situational barriers to intended actions. Judgm. Decis..

[39] Søgaard A.J., Selmer R., Bjertness E., Thelle D. (2004). The Oslo Health Study: The impact of self-selection in a large, population-based survey. Int. J. Equity Health.

[40] Strandhagen E., Berg C., Lissner L., Nunez L., Rosengren A., Torén K., Thelle D.S. (2010). Selection bias in a population survey with registry linkage: Potential effect on socioeconomic gradient in cardiovascular risk. Eur. J. Epidemiol..

[41] Regber S., Novak M., Eiben G., Lissner L., Hense S., Sandström T.Z., Ahrens W., Mårild S. (2013). Assessment of selection bias in a health survey of children and families—The IDEFICS Sweden-study. BMC Public Health.

[42] Australian Bureau of Statistics (2018). Census of Population and Housing: Socio-Economic Indexes for Areas (SEIFA), Australia, 2016. https://www.abs.gov.au/AUSSTATS/abs@.nsf/DetailsPage/2033.0.55.0012016?OpenDocument.

