# Supplementary material for: Pet Owners and Antibiotics: Knowledge, Opinions, Expectations, and Communication Preferences
Source: Antibiotics (Basel). 2021 Oct 29;10(11):1326. doi: 10.3390/antibiotics10111326 (PMC8615269; doi:10.3390/antibiotics10111326)
Supplement: Supplementary file 1 [file antibiotics-10-01326-s001.zip › Document S1 Pet Owner Questionnaire.pdf]

Pet Owner Survey

Start of Block: Welcome and thank you

Q1 Hi there, Aussie Dog or Cat Owner!

Thank you very much for taking 15 minutes out of your day to help me with my PhD research. I really look forward to reading your responses. Every answer gives me a better understanding of what pet owners think and feel about antibiotics. I will use this information to design resources for vets, that help them communicate more effectively with owners about this topic.

Please click on this link and read the participant information document before proceeding.20200818 pet owner pls v2

To move through the survey, **click the arrow buttons** at the bottom of each section. At the end you will be asked whether you'd also like to be interviewed. Don't forget to click the very last arrow button in order to submit your responses.

When you're done, if you would **pass the survey link on** to just one or two (or twenty-five) friends who own a dog or cat, anywhere in Australia, it would help me tremendously!

Warmest thanks,
Ri Scarborough, BVSc (Hons), PhD Candidate, University of Melbourne
(UniMelb human research ethics approval 2057499.1)

End of Block: Welcome and thank you

Start of Block: First, I'd like to know a little bit about you, please.

Q2 **This survey is for Australian residents who own (or have previously owned) a cat or dog.** What is your residential postcode, please?

____

Q3 **And which of these best describes where you live?**

- ○ Capital city, inner urban
- ○ Capital city, outer urban
- ○ Regional city or large town
- ○ Small town
- ○ Rural or remote property not in a town

Q4
What is your gender?
We ask this to check we are getting responses from a broad sample of pet owners. If you prefer not to answer, you can skip this question.

- ○ Male
- ○ Female
- ○ Non-binary

Q5 **What is your age?**
We ask this to check we are getting responses from a broad sample of pet owners. If you prefer not to answer, you can skip this question.

- ○ 20 or under
- ○ 21-30
- ○ 31-40
- ○ 41-50
- ○ 51-60
- ○ 61-70
- ○ over 70

Q6 **What is the highest level of education you have completed?**
We ask this to check we are getting responses from a broad sample of pet owners. If you prefer not to answer, please skip this question.

- ☐ I did not complete primary school
- ☐ Primary school
- ☐ Secondary school certificate
- ☐ Diploma or Certificate
- ☐ Bachelor degree
- ☐ Postgraduate qualification

Q7
Please select the fields in which you have ever:
- undertaken any university study; and/or
- worked
Select all that apply

- [ ] Human health
- [ ] Animal health
- [ ] Agriculture or animal production
- [ ] Scientific research
- [ ] Science education
- [ ] NONE of the above

Q8 **What kind of pet/s do you currently own?**
Select all that apply

- [ ] Dog
- [ ] Cat
- [ ] I don't own a dog or cat at the moment, but I have in the past

End of Block: First, I'd like to know a little bit about you, please.

Start of Block: Block 2

Q9 **When was the last time you took a cat or dog to a vet for ANY of the following: an illness, e.g. diarrhoea, vomiting, fever, coughing, not eating, change in urination a wound or abscess; or another skin or ear problem?**

- ( ) In the last 6 months
- ( ) 6-12 months ago
- ( ) 1-2 years ago
- ( ) More than 2 years ago

Skip To: End of Block If When was the last time you took a cat or dog to a vet for ANY of the following: an illness, e.g.... = More than 2 years ago

Q10 **Now think back *to before* you took your animal to the vet.**
Did you *hope* or *expect* that your pet would get medication for this problem?

◯ Yes

◯ No

◯ Not sure

Display This Question:
If Now think back to before you took your animal to the vet. Did you hope or expect that your pet wo... = Yes

Q11 **In a few words, please explain *why* you hoped or expected your animal would get medication.**

____
____
____
____
____

Q12 **Did your animal get antibiotics for this problem?**
There are many types of antibiotics, and they come in many forms. They are commonly: injected by the vet given by mouth as tablets, capsules, pastes or liquid, or applied to the skin or ear directly as a cream, ointment or lotion

◯ Yes

◯ No

◯ Not sure

Display This Question:
If Did your animal get antibiotics for this problem? There are many types of antibiotics, and they... != Not sure

Q13 **How did you feel about the decision to use (or not use) antibiotics at that time?**

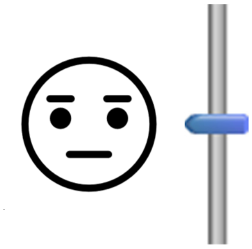

1
2
3
4
5

End of Block: Block 2

Start of Block: Vets can say the same thing in different ways. What's most helpful to you?

Q14 Imagine your vet has examined your unwell pet and determined the he/she probably (or certainly) does NOT need antibiotics. Which of these statements from your vet would make you feel most comfortable with a decision NOT to give antibiotics?
Note: These are similar, but not the same. Pick the one that immediately feels best.

- ◯ Your pet doesn't need antibiotics today, but here are some things we can do to make him/her more comfortable.
- ◯ Here are some things we can do to make your pet more comfortable, but he/she doesn't need any antibiotics today.

Q15 **Which of these statements from your vet would make you feel most comfortable with a decision NOT to give antibiotics?**

- ◯ In my experience, giving antibiotics does not help this to get better any faster.
- ◯ Clinical trials have shown that using antibiotics does not help this to get better any faster.
- ◯ Your pet's immune system can fight this off without antibiotics, if we just give it some time.

Q16 **Which of these statements from your vet would make you feel most comfortable with a decision NOT to give antibiotics?**

- ◯ It's best for your animal not to have antibiotics unless they are really needed, and in this case he/she doesn't need them.
- ◯ Antibiotics can have negative effects on your pet, so it's good that he/she doesn't need them today.
- ◯ It's important that we use antibiotics only when they're needed, and your animal doesn't need them today.

Q17 Which of these statements from your vet would make you feel most comfortable with a decision NOT to give antibiotics?

- ◯ I'm not too worried about your pet's condition at the moment, so I don't think he/she needs antibiotics.
- ◯ Your pet is still eating and drinking, temperature is normal, gum colour is good and the chest is clear, so I am comfortable that he/she doesn't need antibiotics.

End of Block: Vets can say the same thing in different ways. What's most helpful to you?

Start of Block: Block 6

Q18 Imagine your vet has told you that your pet's condition *usually* resolves by itself in about 3 days, but occasionally that doesn't happen and then it might need antibiotics. Which of these statements from your vet would make you feel most comfortable with a decision NOT to give antibiotics today?

- ◯ If it's not getting better in 3 days, please give me a call and I'll arrange some antibiotics for you to pick up.
- ◯ If it's not getting better in 3 days, please come back in to the clinic and we'll re-check your animal, no charge, and decide on treatment then.
- ◯ If it's not getting better in 3 days, please come back in and pick up some antibiotics. I'll leave a note on your electronic file that any of our staff can give those to you, without another consultation.
- ◯ If it's not getting better in 3 days, please come back in and pick up some antibiotics. Here is a prescription that you can bring back to this clinic and give to any member of our team. Then they can give you the antibiotics, without another consultation.

End of Block: Block 6

Start of Block: Block 3

Q19 **Have you ever wanted a vet to give your cat or dog antibiotics, specifically?**

- ◯ Yes, and I have asked for them specifically
- ◯ Yes, and I have hinted that I wanted them, but I have never asked
- ◯ Yes, but I have never hinted or asked
- ◯ Never

Q20 **Have you ever been surprised, disappointed or frustrated when a vet did *not* give your cat or dog antibiotics?**

- ◯ Yes, very
- ◯ Yes, a little
- ◯ Never

Display This Question:
If Have you ever been surprised, disappointed or frustrated when a vet did not give your cat or dog... != Never

Q21 **Why were you surprised, disappointed or frustrated?**
Give as much detail as you can.

____
____
____
____
____

*

Q22 Imagine your vet has said your pet needs antibiotics. There are two choices:

1) the best antibiotic for the job, which is a tablet that needs to be given twice a day for 5 days
2) A single injection lasting 14 days, given by the vet, but it's not the best antibiotic for the job.

Choose UP TO THREE things from this list that are most likely to convince you to choose the tablets over the injection.

- ▢ The tablets are much less likely to create antibiotic-resistant bacteria (superbugs) in your pet
- ▢ The tablets are less than half the price of the injection
- ▢ The tablets are 10% more likely to cure your pet
- ▢ The tablets are much less likely to cause side effects in your pet
- ▢ If the tablets don't work or cause nasty side effects, we can easily stop them and change to a different treatment. But if that happens with the injection, we can't take it out.
- ▢ The tablets are the most responsible choice for public health
- ▢ The tablets are the best choice according to the Australian Veterinary Prescribing Guidelines

End of Block: Block 3

Start of Block: Block 5

Q23 **How much do you agree with the following statements about vets and treatment choices?**

	Strongly DISAGREE	Somewhat disagree	NEUTRAL	Somewhat agree	Strongly AGREE
Vets should make treatment as cheap as possible for owners	○	○	○	○	○
I would be annoyed if my animal was not cured the first time, and I had to go back to the vet	○	○	○	○	○
Vets should make treatment as convenient as possible for owners	○	○	○	○	○
When an animal is sick, sometimes all it needs is some nursing care and time	○	○	○	○	○
If I take a sick pet to the vet, I would be upset if it didn't get any medication	○	○	○	○	○
If my sick pet doesn't get any medication, I feel that I haven't got good value from the vet consultation	○	○	○	○	○
I trust my vet to do the right thing by my animal	○	○	○	○	○
I would probably choose a cheaper treatment option, over a more effective option that was more expensive	○	○	○	○	○
I would probably choose an easier treatment option, over a more effective option that was more work for me	○	○	○	○	○
When my pet is sick, I want my vet to give me a few different treatment options and let me decide	○	○	○	○	○
When my pet is sick, I want my vet to give me one clear recommendation that is the best thing for my pet	○	○	○	○	○
If my pet is sick and the vets says antibiotics *probably* won't help, I would still want my pet to get antibiotics, just in case.	○	○	○	○	○

End of Block: Block 5

Q24 **How much do you agree with the following statements?**

	Strongly DISAGREE	Somewhat disagree	NEUTRAL	Somewhat agree	Strongly AGREE
The risks of antibiotics are so small that they're not worth worrying about	○	○	○	○	○
Antibiotics almost always help a sick animal get better quicker	○	○	○	○	○
All infections, whether viral or bacterial, should be treated with antibiotics	○	○	○	○	○
Giving my pet antibiotics can have a negative effect on its health	○	○	○	○	○
Giving my pet antibiotics could have a negative effect on my health	○	○	○	○	○
Giving my pet antibiotics could have a negative effect on people and animals outside my household	○	○	○	○	○
Bacteria are known to transfer from pets to their owners	○	○	○	○	○
Bacteria are known to transfer from owners to their pets	○	○	○	○	○
Bacteria that are resistant to antibiotics ("superbugs") are a serious problem in Australia	○	○	○	○	○
Vets should only give antibiotics to my pet only when they are really needed	○	○	○	○	○
Vets have a responsibility to protect animal and human public health	○	○	○	○	○
I'd prefer my pet not to receive antibiotics, if they can be avoided	○	○	○	○	○

Q25 **Is there anything else you would like to tell the researchers about your thoughts or experiences with vets, pets and antibiotics?**

____

____

____

____

End of Block: Almost at the end now. Thanks so much for sticking with it!

Start of Block: Block 4

Q26 After completing this survey, some owners will be invited to take part in a 45-minute interview to explore their thoughts and experiences around antibiotic use. The interview will be led by one or two University researchers and will be done online using a secure Zoom meeting, at a time that suits you. The interview will be audio-recorded and the content analysed.

Participants who complete an interview will receive a $20 Coles Myer gift voucher as partial compensation for their time.

Would you like to be considered for an interview?

- ◯ Yes, I am happy to be invited to an interview
- ◯ No, I don't want to be invited to an interview.

Display This Question:
If After completing this survey, some owners will be invited to take part in a 45-minute interview t... = Yes, I am happy to be invited to an interview
