# Supplementary material for: Pet Owners and Antibiotics: Knowledge, Opinions, Expectations, and Communication Preferences
Source: Antibiotics (Basel). 2021 Oct 29;10(11):1326. doi: 10.3390/antibiotics10111326 (PMC8615269; doi:10.3390/antibiotics10111326)
Supplement: Supplementary file 1 [file antibiotics-10-01326-s001.zip › Figure S1 Demographics of respondents excluded from analysis.pdf]

**Category** (% missing data)		n	Proportion	
**Gender** (41%)	**Female**	56	92%	
	Male	5	8%	
	Non-binary	0	0%	
**Age group** (41%)	**30-**	20	33%	
	31-40	9	15%	
	41-50	15	25%	
	51-60	12	20%	
	61+	5	8%	
**Pets owned** (39%)	**Dog(s) only**	28	44%	
	Cat(s) only	14	22%	
	Dog(s) and cat(s)	17	27%	
	Past pets only	4	6%	
**Australian state of residence** (51%)	**VIC**	34	67%	
	NSW	12	24%	
	QLD	1	2%	
	All other states/territories (total)	33	6%	
**Residential area description** (41%)	**Capital city, inner urban**	29	48%	
	Capital city, outer urban	14	19%	
	Regional city or large town	12	20%	
	Small town	6	12%	
**SEIFA quintile of residential area** (49%)	1 - least advantaged	1	2%	
	2	5	9%	
	3	12	23%	
	4	6	11%	
	**5 - most advantaged**	29	55%	
**Highest education** (41%)	School	6	10%	
	Diploma or Certificate	19	31%	
	**Bachelor degree**	23	38%	
	Postgraduate qualification	13	21%	
**Fields of university study or work** (0%)	Animal production or agriculture	3	3%	
	Animal health	9	9%	
	**Human health**	20	19%	
	Scientific research	1	1%	
	Science education	1	1%	
	None of the above	70	67%	

Figure S1: Demographics of respondents excluded from analysis
